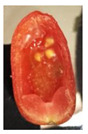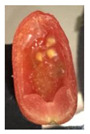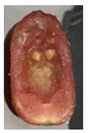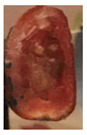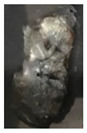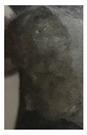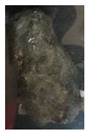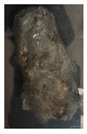# Corn Starch/Chitosan Nanoparticles/Thymol Bio-Nanocomposite Films for Potential Food Packaging Applications

**DOI:** 10.3390/polym13030390

**Published:** 2021-01-27

**Authors:** Siti Hajar Othman, Nur Fitrah Liyana Othman, Ruzanna Ahmad Shapi’i, Siti Hajar Ariffin, Khairul Faezah Md. Yunos

**Affiliations:** 1 Department of Process and Food Engineering, Faculty of Engineering, Universiti Putra Malaysia, Serdang 43400, Selangor, Malaysia; fitrahliyanafl@gmail.com (N.F.L.O.); ruzannashapii91@gmail.com (R.A.S.); hajarariffin@upm.edu.my (S.H.A.); kfaezah@upm.edu.my (K.F.M.Y.); 2 Institute of Advanced Technology, Universiti Putra Malaysia, Serdang 43400, Selangor, Malaysia

**Keywords:** biopolymer, chitosan nanoparticles, film, food packaging, nanocomposite, starch, thymol

## Abstract

This work aims to develop corn starch/chitosan nanoparticles/thymol (CS/CNP/Thy) bio-nanocomposite films as potential food packaging materials that can enhance the shelf life of food. CS/CNP/Thy bio-nanocomposite films were prepared by the addition of different concentrations of thymol (0, 1.5, 3.0, 4.5 *w*/*w*%) using a solvent casting method. The resulting films were characterized in terms of optical, mechanical, and water vapor permeability (WVP) properties. The addition of thymol was found to reduce the tensile strength (TS), elongation at break (EAB), and Young’s modulus (YM) of the films. Generally, the increment in the concentration of thymol did not significantly affect the TS, EAB, and YM values. The addition of 1.5 *w*/*w*% thymol increased the WVP of the films but the WVP reduced with the increase in thymol concentrations. CS/CNP/Thy-3% bio-nanocomposite films demonstrated the potential to lengthen the shelf life of cherry tomatoes packed with the films, whereby the cherry tomatoes exhibited no significant changes in firmness and the lowest weight loss. In addition, no mold growth was observed on the sliced cherry tomatoes that were in direct contact with the films during 7 days of storage, proving the promising application of the films as active food packaging materials.

## 1. Introduction

Plastic material is used extensively for food packaging applications due to its low cost, lightweight nature, high performance, and good processability. However, conventional plastic packaging materials are usually derived from non-renewable petroleum resources that exhibit non-degradable properties [1], thus excessive amount of plastic usage may result in the accumulation of municipal solid waste which can negatively affect environmental sustainability [2]. This issue can be overcome by finding alternative food packaging materials such as those made from biopolymers. Renewable biopolymers such as polysaccharides, proteins, polyesters, and their composites, which are derived from plant and animal resources, can be used to replace the non-degradable petrochemical-based plastics. 

Among the many biopolymers, starch, which falls under the polysaccharide group, is reliable to be developed into food packaging materials due to its environmentally friendly nature, flexibility, transparency, thermoplastic properties, and low cost. In particular, corn plant is the primary source of native starches that is commercially available, and more than 85% of starch production in the world is extracted from corn trees, thus producing corn starch [3]. Corn starch has the highest amylose content among other types of native starches and is responsible for good film-forming properties. However, starch-based films exhibit some limitations such as brittleness and exhibit poor mechanical and barrier properties [4]. These limitations can be encountered by adding plasticizers such as glycerol and blending starch with other natural polymers, hydrophobic substances, and/or antimicrobial compounds such as chitosan, which can establish the functional properties of the starch-based material [5,6,7]. Chitosan is suitable to be used for food packaging applications due to its non-toxic, biodegradable, biofunctional, biocompatible, and antimicrobial properties. Subsequently, good adherence between starch and chitosan can be attained with the combination of hydrogen bonding; opposite charge attraction between chitosan cations and negatively charged surface of starch film [8]. 

An advancement of nanotechnology revealed that the incorporation of nanosized fillers into biopolymer to produce bio-nanocomposite can further improve the properties of biopolymers whereby the large surface area of the nanosized fillers leads to large interphase or boundary area between the biopolymer matrix and fillers. The nanosized fillers act as a reinforcement in the bionanocomposite [9]. According to Shapi’i et al. [10], bulk chitosan can be synthesized into nanoparticles, namely, chitosan nanoparticles (CNPs) that have dimensions ranging from 1 to 100 nm. The incorporation of CNP may enhance the mechanical and barrier properties of the biopolymers [1]. 

Despite the advantages, CNP only inhibits bacteria that are in direct contact with the CNP, thus limiting the application of the biopolymer as potential food packaging material. The addition of other active agents, such as thymol, that are volatile may also contribute to the synergistic effect of the active properties of the films, particularly the synergistic antimicrobial effect. The incorporation of thymol into the food packaging as a natural antimicrobial agent tends to have great potential in improving the shelf life of perishable food products such as tomatoes. Thymol (2-isopropyl-5-methyl phenol) is the main phenolic monoterpene found in the essential oils extracted from plants such as thyme and oregano which belong to the Lamiaceae family [11]. However, the addition of different concentrations of thymol on the biopolymer films may affect the optical, mechanical, and barrier properties of the films. The main reason was due to the different concentrations of hydrophobic constituents added to the film matrix that will affect the hydrophilic/hydrophobic balance of the film [12]. 

To the best of our knowledge, no work has been carried out to investigate the optical, mechanical, and barrier properties of corn starch (CS) films incorporated with both CNP and thymol (Thy) or CS/CNP/Thy bio-nanocomposite films. The studies in the literature mostly reported the effect of thymol addition on starch films without the additional incorporation of nanosized fillers such as CNP. For instance, Ghasemlou et al. [13] investigated the effects of the addition of two types of essential oils that are Zataria multiflora Boiss (ZEO) or Mentha pulegium (MEO) in corn starch films; Nordin et al. [14] explored the properties of corn starch films incorporated with glycerol and thymol; Davoodi et al. [15] studied the properties of potato starch films incorporated with polysorbate-thymol. Moreover, no work has been carried out to investigate the potential application of the CS/CNP/Thy bio-nanocomposite films to be used as food packaging in the context of the shelf life of food packed with the films. 

**Figure 1:**
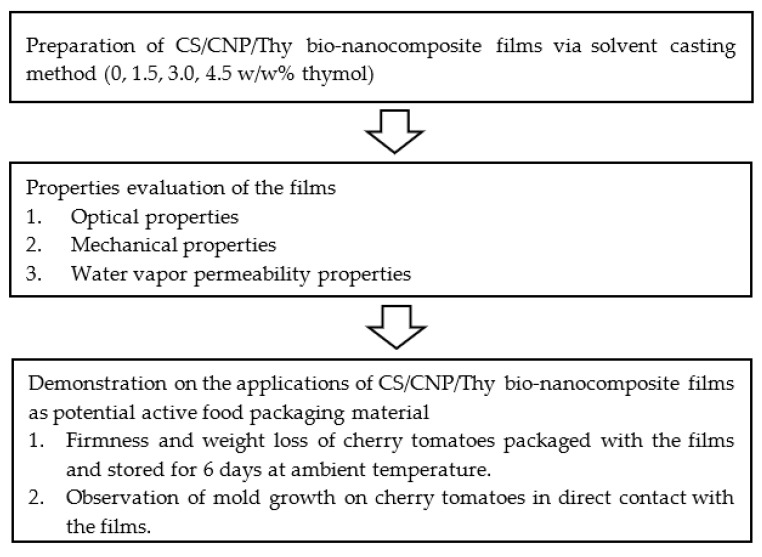
Summary of procedures for the development of corn starch/chitosan nanoparticles/thymol (CS/CNP/Thy) bio-nanocomposite films as potential active food packaging material.

In the present study, corn starch films with the incorporation of CNP and different concentrations of thymol, which is a novel formulation, were prepared via a solution casting method. The effects of thymol concentrations on the optical, mechanical, and water vapor permeability properties of the films were investigated. The potential application of the films as food packaging material was demonstrated on the shelf life of cherry tomatoes packed with the films in terms of firmness, weight loss, and observation of mold growth. Cherry tomato (Solanum Lycopersicum Mill.) is chosen as the food for shelf-life study because tomato is one of the widely grown vegetable crops, the second most important source of nourishment after potatoes for the world’s population with a total production of around 160 million tons per year [16]. In addition, tomato is one of the perishable fruits, and the ripeness changes continuously after harvesting. The typical problem related to most of the perishable food is the microbial growth that affects the food quality, alters textural property, and adversely affects the color and nutrition of the food product. Figure 1 summarizes the procedures for the development of CS/CNP/Thy bio-nanocomposite films as potential active food packaging material.

## 2. Materials and Methods 

### 2.1. Materials

Chitosan (low molecular weight: 50 kDa, viscosity: 20–300 cP in 1% *w*/*v* of acetic acid, deacetylation: 75–85%) and sodium tripolyphosphate (TPP) were purchased from Sigma-Aldrich, Saint Louis, MO, USA. Corn starch (33% amylose, 67% amylopectin), acetic acid, Tween 80, glycerol, thymol, sodium hydroxide (NaOH), sodium bromide (NaBr), magnesium nitrate (MgNO_3_), calcium chloride (CaCl_2_), paraffin wax, bee wax, and isopropyl alcohol were purchased from R&M Marketing, Surrey, UK. 

### 2.2. Cherry Tomato Samples

Cherry tomato samples packed in vented plastic clamshell containers were purchased from Kea Farm, Cameron Highlands, Pahang, Malaysia. Cherry tomatoes of the same red ripe maturity stage (stage 6) as well as of about the same size were sorted for the analysis and were stored in a chiller (Temperature: 5 °C) before being used for analysis the next day. 

### 2.3. Synthesis of Chitosan Nanoparticles (CNP)

CNP was first synthesized via the ionic gelation process of chitosan and TPP. All parameters were controlled based on the optimum parameters that produced the most stable and smallest-sized CNP as reported by Shapi’i et al. [17]. First, chitosan solution (15% *w/w* of solid corn starch) was prepared by dispersing 0.45 g chitosan flakes into 50 mL aqueous acetic acid solution (1% *v/v*) using a magnetic stirrer (FAVORIT HS0707V2, Jakarta, Indonesia) for 30 min. Then, the pH of the solution was adjusted to pH 4.6 using 10% *v/v* NaOH.

TPP solution was prepared according to the ratio of chitosan to TPP (5:1) by dissolving 0.09 g of TPP powder in 50 mL distilled water. CNP was spontaneously obtained upon the addition of 50 mL of TPP solution drop by drop to the 50 mL chitosan solution under vigorous magnetic stirring at room temperature (25 °C) for 30 min. Then, a centrifuge (Universal 320, Tuttlingen, Germany) was used to disperse the CNP emulsion at 9000 rpm for 30 min. The centrifugation process was performed two times for washing, thus producing the CNP in gel-like suspension. 

### 2.4. Preparation of the Films

An amount of 3 g of corn starch (CS) was dispersed in 50 mL distilled water–glycerol solution where the amount of glycerol was fixed to 0.75 g (25% *w/w* of solid CS). Then, the solution was heated with continuous stirring using a magnetic stirrer until gelatinized completely at 85 °C [14]. Meanwhile, the CNP solution was prepared by dissolving 3 g CNP gel-like suspension with 50 mL distilled water and the solution was sonicated using an ultrasonic probe (QSonica, Newtown, CT, USA, 500 W, 20 kHz) for 15 min at 50% amplitude. The CS solution was then cooled down to 40 ± 2 °C before being mixed with the CNP solution using a magnetic stirrer to produce CS/CNP bio-nanocomposite film solution. 

Table 1 shows the different amounts of thymol added to the CS/CNP bio-nanocomposite film solutions to produce CS/CNP/Thy bio-nanocomposite film solutions with different concentrations of thymol. The different amounts of thymol, 275 µL of Tween 80, and CNP solution were mixed to produce 50 mL thymol/CNP solution using a magnetic stirrer. The CS/CNP/Thy bio-nanocomposite film solution was prepared by mixing the thymol/CNP solution and 50 mL gelatinized CS solution and stirred for 10 min using a magnetic stirrer. After mixing, the solution was subjected to sonication for 5 min at 50% amplitude using an ultrasonic probe to produce a homogenous solution. An amount of 40 mL of the mixture solution was poured into an acrylic petri dish (diameter: 14 cm) and left to dry in the air-conditioned room (25 °C) for 48 h on the flat table. A corn starch film without the addition of CNP was also prepared as the film control. 

**Figure 2:**
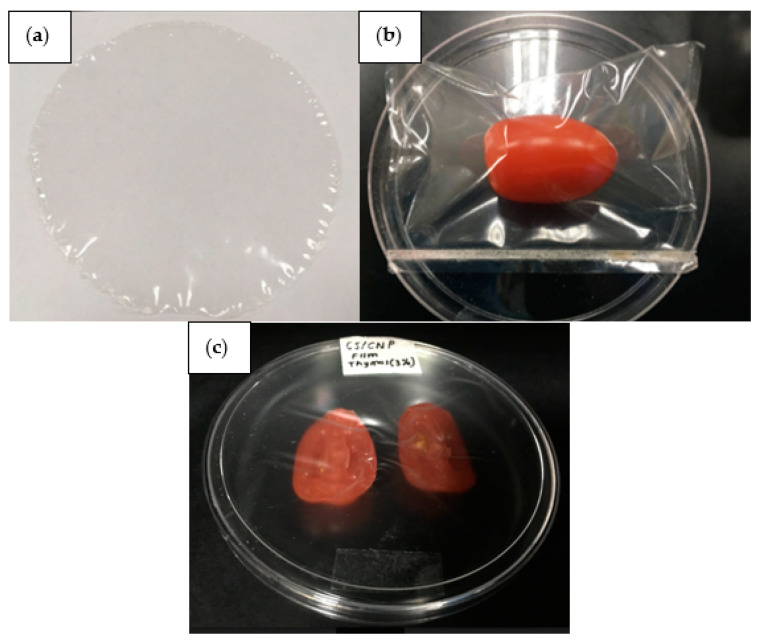
(**a**) Starch/CNP/Thy bio-nanocomposite film; (**b**) cherry tomato that was packed using the produced film; (**c**) sliced cherry tomato that was placed in the petri dish, in direct contact with the film for the shelf-life observation study.

After drying, the film was peeled off from the petri dish and conditioned in a desiccator containing saturated MgNO_3_ solution (relative humidity (RH): 51%, Temperature: 30 °C). Figure 2a shows a transparent and smooth film produced after peeled off from the petri dish. The thickness of the film was measured using a digital micrometer (Mitutoyo, Kanagawa, Japan) at five random positions around the film. The average values of the thickness were used to calculate the tensile strength (TS), elongation at break (EAB), and Young’s modulus (YM), opacity, and water vapor permeability (WVP). 

### 2.5. Characterization on Optical Properties of the Films

The optical properties of the films were determined based on color and opacity of the films. The color of the films was determined using a color spectrophotometer (HunterLab, Ultrascan Pro, Reston, VA, USA) by measuring the CIELAB coordinates of L*, a*, and b*, whereby L* = 0 (black) to L* = 100 (white), −a* (greenness) to +a* (redness), and −b* (blueness) to +b* (yellowness). The total color difference (ΔE) was calculated using Equation (1):

∆E = ([∆L]^2^ + [∆a]^2^ + [∆b]^2^)^1/2^ (1)

where ΔL* is the differential value of L* of the films, Δa* is the differential value of a* of the films, and Δb* is the differential value f b* of the films. The differentials were calculated between the control film and film samples.

The light transmittance of the film samples was measured using the same color spectrophotometer at a wavelength range of 200–700 nm. Then, the opacity values of the films were calculated using the following Equation (2):

Opacity = A_600_/L (2)

where A_600_ is the absorbance value at 600 nm and L is the thickness of the film (mm).

### 2.6. Mechanical Properties

The mechanical properties of the films were determined based on tensile strength (TS), elongation at break (EAB), and Young’s modulus (YM) values of the films measured using a texture analyzer (TA.XT2 Stable Micro Systems, Surrey, UK) according to American Society for Testing and Materials (ASTM) D882 [18]. Film strips (100 mm × 15 mm) were cut from each pre-conditioned sample film and placed between the grips. Initial grip separation and test speed were set to 60 mm and 0.5 mm s^−1^, respectively. 

### 2.7. Water Vapor Permeability 

The barrier properties of the films were determined in terms of water vapor permeability (WVP). The determination of WVP was carried out using a modified dry cup method according to ASTM E96 [19]. The films were cut into circular shapes using a cutter (diameter: 7 cm) and placed on the cup containing 10 g of CaCl_2_ (RH = 0%). The mixture of paraffin wax and bee wax (8:2) was heated using the magnetic stirring hotplate until the mixture melted. The mixture of waxes was then poured around the ring of the cup, which acted as the sealant. 

To maintain the surrounding humidity of the cups, each cup was stored in a desiccator containing saturated MgNO_3_ to provide a constant RH of 51% at 30 °C. A digital temperature humidity meter (Proskit NT-312, Penang, Malaysia) was used to monitor the relative humidity and temperature. Changes in the weight of the cup were recorded every 24 h for 10 days and the weight was plotted against time to obtain the weight loss versus time graph. 

The water vapor transmission rate (WVTR) was calculated based on Equation (3):

WVTR = (W/t)/A(3)

where W/t is the slope of weight changes versus time graph (g/h) and A is the transmission area of the film (28 ${\mathrm{cm}}^{2}$). 

The water vapor permeability (WVP) was calculated based on Equation (4):

(4) $$\mathrm{WVP}\;=\;(\mathrm{WVTR}\;\times \;L)/(P_{1}-{\;P}_{2})$$

where L is the average of film thickness (mm), P_1_ is the water vapor partial pressure in desiccator at RH = 51%, 21.64 × 10^5^ Pa, and P_2_ is the water vapor partial pressure in the cup at RH = 0%, 0 Pa.

### 2.8. Demonstration on the Application of the Films as Food Packaging Materials

The potential application of the films as food packaging material was demonstrated on the cherry tomatoes. The cherry tomatoes were washed under running tap water and air dried to remove the excess moisture. Then, the cherry tomatoes were packed in the films (dimension: 10 cm^2^) and sealed using a sealer (SF-200, Penang, Malaysia). The quality of the cherry tomatoes, in terms of firmness and weight loss, was analyzed on days 0, 3, and 6 of storage at ambient temperature (25 °C). Figure 2b shows the image of the cherry tomato that has been packed using the films and sealed using a sealer. The shelf life of the cherry tomatoes was determined in terms of firmness, weight loss, and through observation.

#### 2.8.1. Firmness

The firmness of the cherry tomatoes was determined using a texture analyzer (TA.XT2 Stable Micro Systems, Surrey, UK) by measuring the force required to make a predetermined piercing using a 2.5 mm diameter flat-tipped cylindrical probe and 50 kg cell. The cherry tomato was compressed by the probe to a 2 mm penetration depth at a speed of 2 mm/sec on days 0, 3, and 6 of storage. 

#### 2.8.2. Weight Loss

The weight loss of the cherry tomatoes was calculated using Equation (5):

(5) $$\mathrm{Weight}\;\mathrm{loss}=\frac{\mathrm{IW}-\mathrm{FW}}{\mathrm{IW}}\;\times \;100$$

where IW is the initial weight of cherry tomato (g), FW is the final weight of cherry tomato at days 0, 3, and 6 (g).

#### 2.8.3. Shelf-Life Observation

For the shelf-life observation of cherry tomatoes, the films were put in direct contact with the cherry tomatoes. A petri dish and a knife were first sanitized using alcohol (isopropyl alcohol) to avoid contamination. Then, the films were cut according to the size of the petri dish lid. A film was placed inside the lid. Subsequently, a cherry tomato was cut into half and placed into the petri dish. The lid that has previously been placed with the film was then used to close the petri dish. Figure 2c shows the sliced cherry tomato that was placed in the petri dish, in direct contact with the film. The sample was stored at ambient temperature (25 °C). 

Changes in the appearance of the cut cherry tomatoes as well as mold growth on the surface of the cut cherry tomatoes were recorded by taking the images of the samples daily for 7 days using a camera phone (iPhone 11 5SE, Foxconn, Zhengzhou, China). The camera phone was placed above the position of the samples to capture the images of the samples from the top. The images were taken in the same conditions of natural light, camera type, camera settings (auto mode, no flash, high dynamic range (HDR) off), height (25 cm), and angle. 

### 2.9. Statistical Analysis

The statistical analyses of the obtained experimental results were performed by analysis of variance (ANOVA) using Minitab 17 software (Minitab Inc., State College, PA, USA). Mean comparisons were conducted using Tukey’s test at a 0.05 level of significance (*p* < 0.05). 

## 3. Results and Discussion

### 3.1. Optical Properties

**Figure 3:**
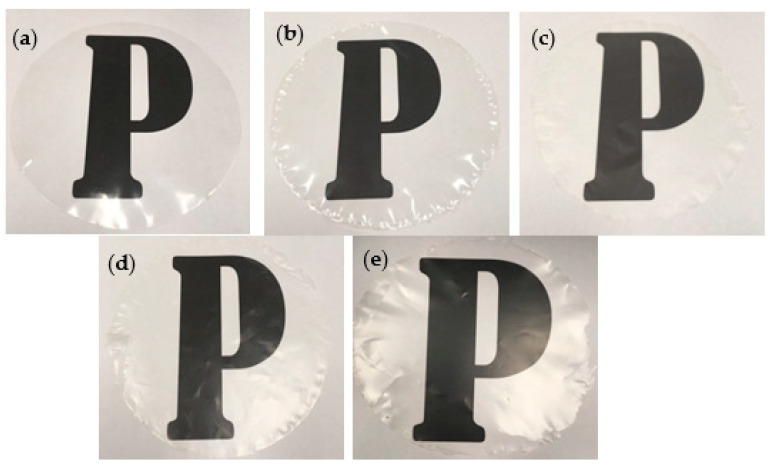
Appearance of the films on alphabet of “P”; (**a**) neat CS film; (**b**) CS/CNP; (**c**) CS/CNP/Thy 1.5%; (**d**) CS/CNP/Thy 3%; (**e**) CS/CNP/Thy 4.5% bio-nanocomposite films.

The L*, a*, b*, ΔE, and opacity of the films were tabulated in Table 2. Based on Table 2, the addition of thymol reduced slightly the L* values and the reduction became more pronounced at a higher concentration of thymol (4.5 *w/w*%). This indicates a slight decrease in lightness due to the existence of thymol. Based on the work by Nordin et al. [14], the L* values of CS films decreased with the addition of thymol due to the intrinsic color of thymol, which is a white crystalline substance that caused the films to become slightly darker. Their work revealed that the L* value for CS films (33.0) added with thymol was much lower than the findings in this study (≈93.0). The high L* values of all the films produced in this study were most probably due to the addition of CNP which increased the glossiness of films, hence improving the appearance of the films [20]. The high glossiness appearance of the films can be observed clearly from Figure 3, whereby Figure 3 shows the appearance of the films on alphabet “P”. A similar trend of L* values was also obtained when the different concentrations of Thymus kotschyanus essential oil were added into starch/chitosan composite films [21]. Furthermore, Wang et al. [22] reported that both neat CS film and CS film incorporated with orange-peel oil and zein nanocapsules exhibited high L* values, consistent with the findings in this work.

Table 2 shows that there was no significant change in the a* values for all the films. The small negative a* values indicated that the films have a tinge of green color. Nonetheless, the a* values were very small, and the tinge of green color was non-noticeable as can be seen from Figure 3. The films also have a tinge of yellow color which was proven from the small positive b* values due to the addition of CNP into the starch matrix. Maillard reaction between amino and hydroxyl groups of chitosan produced insoluble melanoidin compounds that led to a tinge of yellow color films. The color was absorbed in wavelengths near the yellow region, due to the presence of CNP in the films [23]. When a higher concentration of thymol (4.5*w/w*%) was added to the CS/CNP films, the b* value increased slightly because the high concentration of thymol incorporated in the starch matrix induced the light scattering on the surface of the films [24]. The light scattering played a role in the yellow pigment of the films due to wide-angle scattering; thus, more yellow light can be transmitted. However, the b* values were very small and the change in yellow color was non-noticeable as can be observed from Figure 3.

Furthermore, there was a slight increment in ΔE values (*p* < 0.05) when thymol concentrations were increased from 0 to 4.5*w/w*%. This trend was expected due to the changes in L*, a*, and b* values as discussed earlier. Additionally, it can be seen from Table 2 that the addition of a high concentration of thymol (4.5*w/w*%) resulted in a slight increase in the opacity value of the films, which indicates lower transparency of the films. Lozano-Navarro et al. [25], who incorporated chitosan-starch films with natural extracts (oregano, blueberry, and beetroot), and Nordin et al. [14], who produced CS films incorporated with thymol and glycerol, reported that the opacity of their films increased slightly with the addition of the extracts and thymol into the film matrix. Zhong et al. [12] reported that the opacity of peanut protein isolate (PPI) films increased slightly with the increase in thymol concentrations due to the presence of polyphenols in the films. The lipophilic compound inside thymol may hinder the light transmittance and caused light scattering, thus increased the opacity. However, the increment in opacity value found in this study was very small and, when observed from Figure 3, the changes in opacity or transparency of the films were almost non-noticeable. 

### 3.2. Mechanical Properties 

**Figure 4:**
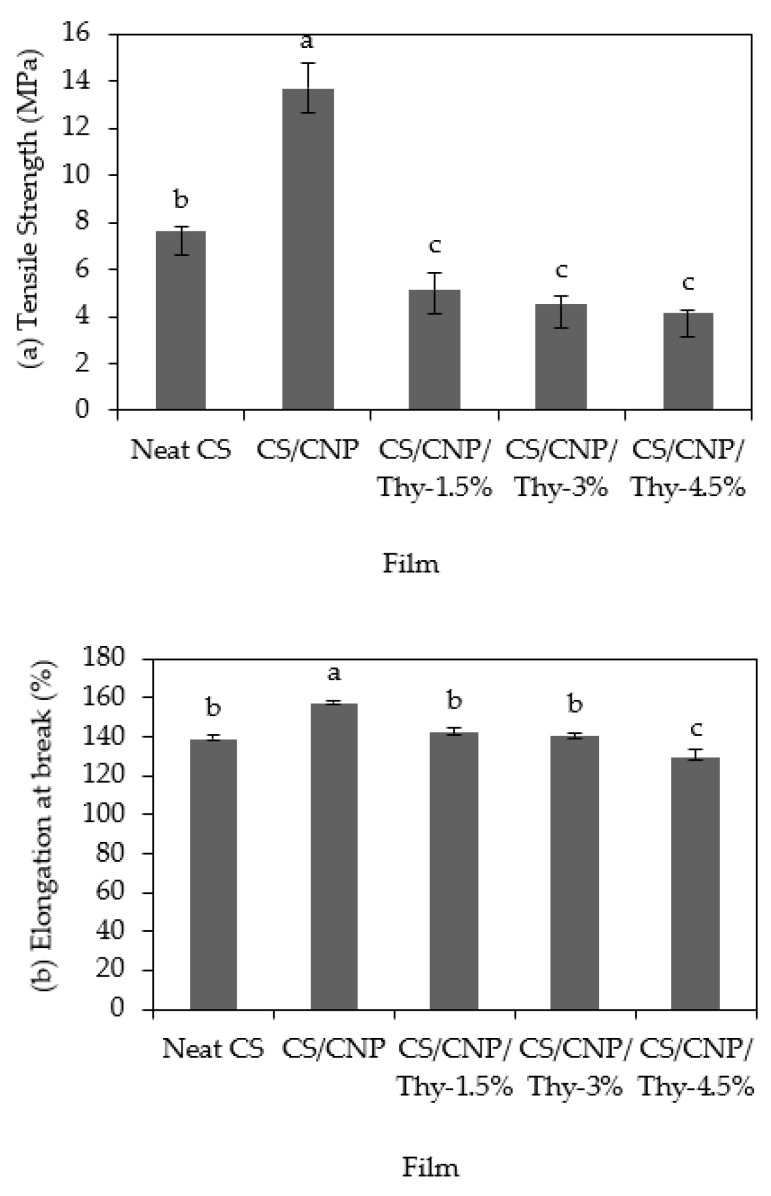

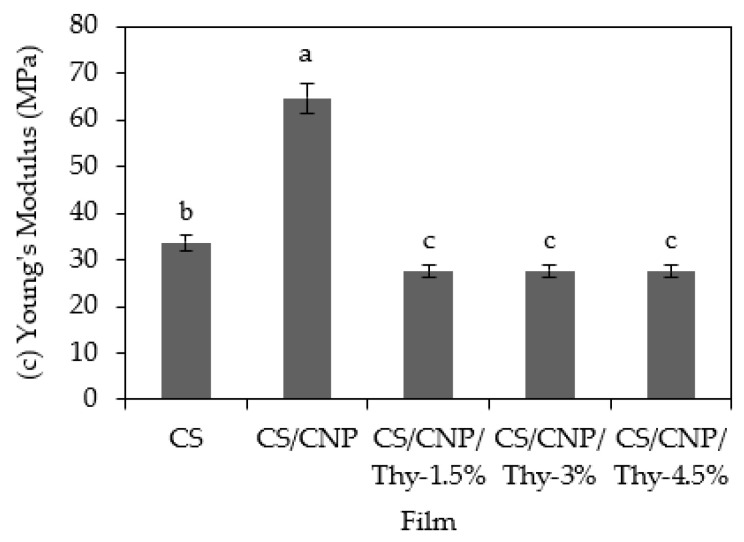
(**a**) Tensile strength (TS); (**b**) elongation at break (EAB); (**c**) Young’s modulus (YM) of the neat CS film, CS/CNP, and CS/CNP/Thy bio-nanocomposite films incorporated with different concentrations of thymol. Different letters in the same graph indicate a statistically significant difference (*p* < 0.05).

Figure 4 shows the TS, EAB, and YM of the neat CS films, CS/CNP bio-nanocomposite films, and CS/CNP/Thy bio-nanocomposite films. Figure 4a shows that neat CS films exhibit poor TS but the incorporation of CNP into the CS films increased the TS of the films. According to Shapi’i et al. [1], the incorporation of chitosan, especially in nanosized form or CNP, has the potential to improve the mechanical properties of starch films. The tiny size and regular shape of the CNP due to the crosslinking of chitosan and TPP during the ionic gelation process facilitated the CNP to fill in the empty spaces between the matrix of the starch [26]. The large surface area of CNP exposed to the starch increased the intermolecular interactions between the starch and CNP. This brought the distances of adjacent starch chains closer, leading to an increase in the density of the starch films. The films became more compact, strong, and thus higher resistant to mechanical stress [27].

Figure 4a also shows the effect of thymol concentrations on the TS of CS/CNP/Thy bio-nanocomposite films. The addition of 1.5*w/w*% of thymol into the films caused a significant reduction in TS from 13.7 MPa for CS/CNP bio-nanocomposite films to 5.1 MPa for CS/CNP/Thy 1.5% bio-nanocomposite films, which was a 63% reduction. However, when the concentration of thymol was increased from 1.5 to 4.5*w/w*%, no significant change (*p* > 0.05) was observed for the TS of the films. The interaction between the starch biopolymer matrix and phenolic compounds resulted in the heterogeneity of the film structure, thus reducing the TS of the films. A similar trend of findings was revealed by Ramos et al. [28] where they found a slight modification of TS when thymol was incorporated into the polypropylene films. The reduction in TS was attributed to the complex structures formed between the lipids from the phenolic compounds and the starch polymers which reduced the cohesion of the starch network forces, thus decreasing the films’ resistance to breakage [29]. The addition of thymol resulted in the lowered interaction between biopolymer monomers and hindered polymer chain-to-chain interactions and reduced cross-linking.

This finding is also consistent with the work of Nordin et al. [14] who incorporated thymol and glycerol in CS films and found a decrease in TS by 96% compared to neat CS films. A similar trend was also observed by Ghasemlou et al. [15] who investigated the TS of starch films incorporated with ZEO or MEO. Tawakkal et al. [30] reported that polylactic acid (PLA) composites incorporated with 30% *w/w* untreated kenaf or treated kenaf fibers with 0, 5, and 10% *w/w* thymol loadings experienced a reduction in TS values. They reported that a localized plasticizing effect between the PLA and the thymol occurred, which facilitated thymol molecule diffusion into the bulk of the matrix between the PLA chains. In this work, the addition of thymol interfered with the interaction between the polymer matrix and the CNP in the presence of the applied stress and subsequently reduced the TS. 

The EAB determines the elasticity of the films and the potential of the films to stretch. Figure 4b shows that the EAB of neat CS films increased from 139 to 157% with the addition of CNP into the films. This was because most of the empty spaces within the starch matrix were filled up with CNP and formed strong hydrogen bonding which increased both TS and EAB. Moura et al. [31] found that the addition of 40% *w/w* CNP increased the EAB of neat carboxymethyl cellulose films. They reported that the improvement of the films’ flexibility was attributed to the optimum particle size of CNP added into the films. A similar trend in the results was achieved when CS film and cassava film were added with chitosan [8]. The authors found that the addition of chitosan in both starch films exhibited higher EAB values, 108% and 146%, respectively, compared to the control films. This was due to the existence of synergic compatibility between starch and chitosan after film formation. When nanoparticles were presented, the number of interface defects in biopolymer matrix reduced, and this led the molecular movement of CNP molecules. Thus, the deformation capacity of polymeric matrix upon application of tension deformation was promoted, which allowed the films to have a higher elongation capacity until their rupture [32]. 

Meanwhile, the incorporation of thymol at different concentrations (1.5 and 3*w/w*%) did not significantly affect the EAB of the films. However, the addition of 4.5*w/w*% thymol to the CS/CNP/Thy bio-nanocomposite films caused a slight reduction in the EAB, whereby EAB reduced from 140% to 129% for neat CS films and CS/CNP/Thy-4.5% bio-nanocomposite films, respectively. As found by Robledo et al. [33] and Nordin et al. [14], the addition of thymol at a certain concentration formed a non-miscible phase in the starch matrix that caused segregation of the starch chains. Hence, the chain mobility reduces, and this causes a reduction in the elasticity of the film. In this work, concentrations of 1.5 and 3*w/w*% might be a bit low to affect the EAB values of the films, but 4.5*w/w*% resulted in the decrease in the EAB of the CS/CNP/Thy bio-nanocomposite films due to a sufficient amount of thymol causing segregation of the starch chains. A similar finding was obtained by Altiok et al. [34], whereby the increment of thyme oil concentrations caused a reduction in EAB of chitosan film. They also stated that the decrement was caused by the increase in pore size and porosity of the films.

Young’s modulus indicates the stiffness or rigidity of the film and a larger value correlates to a more rigid material [35]. Figure 4c shows the effects of thymol addition on the YM of the films. Initially, the addition of CNP into the neat CS film increased significantly the YM of the films from 33.7 to 63.6 MPa. This finding is consistent with the TS value obtained earlier, whereby the increment in TS value indicates an improvement in the rigidity of the films, hence stiffer films, and thus increment in the YM value. A similar trend of results was achieved by Akter et al. [36], whereby the chitosan added into starch films acted as a reinforcing agent in the films, thus increased the YM value. However, this finding was opposite to the finding obtained by Szymańska-Chargot et al. [37], whereby YM values reduced when chitosan was added to the carrot cellulose nanofiber films. Pelissari et al. [35] also found that YM values decreased with the addition of 5% *w/w* of chitosan to the cassava starch film. They explained that chitosan acted similarly to plasticizer and enhanced the ductility when incorporated in a starch network due to the direct interactions and the reduction in proximity between starch chains.

Figure 4c also shows that the YM values decreased significantly with the addition of thymol. Nonetheless, there was no significant change of YM when thymol concentrations were varied from 1.5 to 4.5% *w/w*. A similar trend of results was reported by Villegas et al. [38] whereby a reduction in YM values was observed when thymol was added to PLA. This finding was related to the plasticizing effect of thymol that leads to the change of material stretchability. The addition of thymol resulted in the lowered interaction between the starch monomers and retarded the polymer chain-to-chain interactions and minimized cross-linking [39]. Hence, the decrease in rigidity of films was gained due to the increment of elasticity and low YM values. Pelissari et al. [35] found that the YM of cassava starch–chitosan films increased with the addition of oregano essential oil. This result was relevant to this study because oregano essential oil contained phenolic compounds that were mainly comprised of thymol [40]. Khairuddin et al. [41] also found that the incorporation of 1.5 to 2.5% *w/w* thymol caused the YM decrement of hydroxyethyl cellulose/wheat-starch-based films. They supported their findings by the plasticizing effect caused by the addition of thymol to the polymer matrix. As a result, the ductile properties of the films increased, which was due to the changes in the materials’ crystallinity. 

### 3.3. Water Vapor Permeability

**Figure 5:**
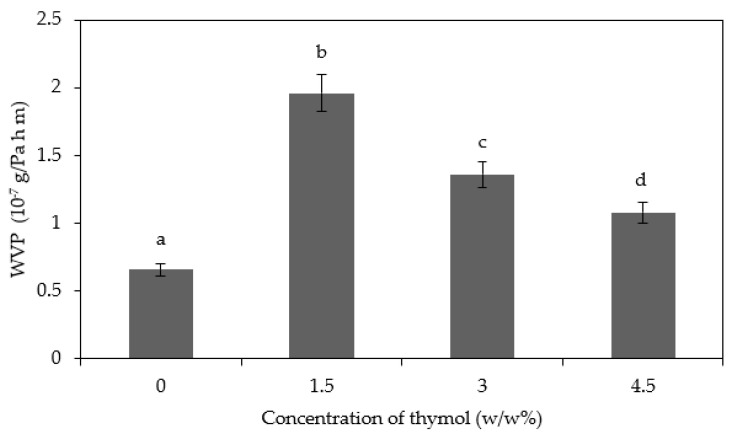
Water vapor permeability (WVP) of the CS/CNP/Thy bio-nanocomposite films incorporated with different concentrations of thymol. Different letters in the same graph indicate a statistically significant difference (*p* < 0.05).

The main function of food packaging is often to avoid or at least to decrease moisture transfer between the food and the surrounding atmosphere. Therefore, the WVP of food packaging material should be as low as possible. From Figure 5, it can be seen that the WVP of CS/CNP bio-nanocomposite films increases from 0.66 × 10^–7^ to 1.96 × 10^–7^ g/Pa h m with the addition of 1.5*w/w*% thymol. A similar trend of results was obtained by Wu et al. [42], whereby the incorporation of 3 wt. % thymol into neat PLA/polycaprolactone (PCL) films resulted in the increment of WVP. They reported that the increment was due to the addition of thymol that increased the average pore size of the films. They observed many voids present in the PLA/PCL/Thy films, through scanning electron microscopy (SEM) images, whereby the voids permitted more water vapor to transfer through the films. Similarly, Kavoosi et al. [39] reported that the incorporation of thymol caused a significant increase in the WVP of gelatin films. The addition of thymol hindered the polymer chain-to-chain interactions and minimized the cross-linking that existed within the biopolymer matrix, thus increasing WVP due to the disruption of the chain matrix. 

The addition of higher concentrations of thymol (3 and 4.5*w/w*%) reduced the WVP due to the hydrophobicity of the thymol [39]. The decrement in the WVP of the films was due to the hydrogen and covalent interactions between the starch network and the polyphenolic compounds, whereby the interactions limited the availability of hydrogen groups to form hydrophilic bonds with water and, subsequently, led to a decrease in the films’ affinity for water [43]. A similar trend of results was reported by Zhong et al. [12] that incorporated thymol into a modified PPI film. They found that the hydrophobic nature of thymol disrupted the hydrophilic/hydrophobic balance of the films, hence decreased the WVP. Thymol also enhanced the hydrophobic nature of the films, thus limiting water vapor penetration through the films [44]. Besides, Pellisari et al. [33] analyzed that the ratio of the hydrophilic–hydrophobic component determined the rate of water vapor transfer which mostly happens at the hydrophilic side. Therefore, increasing the ratio of hydrophobic could develop barrier properties. Other studies also revealed that the addition of essential oils and thymol improved the barrier properties of starch film and gelatin films, respectively [13,39]. 

### 3.4. Demonstration on the Application of CS/CNP/Thy Bio-Nanocomposite Films as Food Packaging

**Figure 6:**
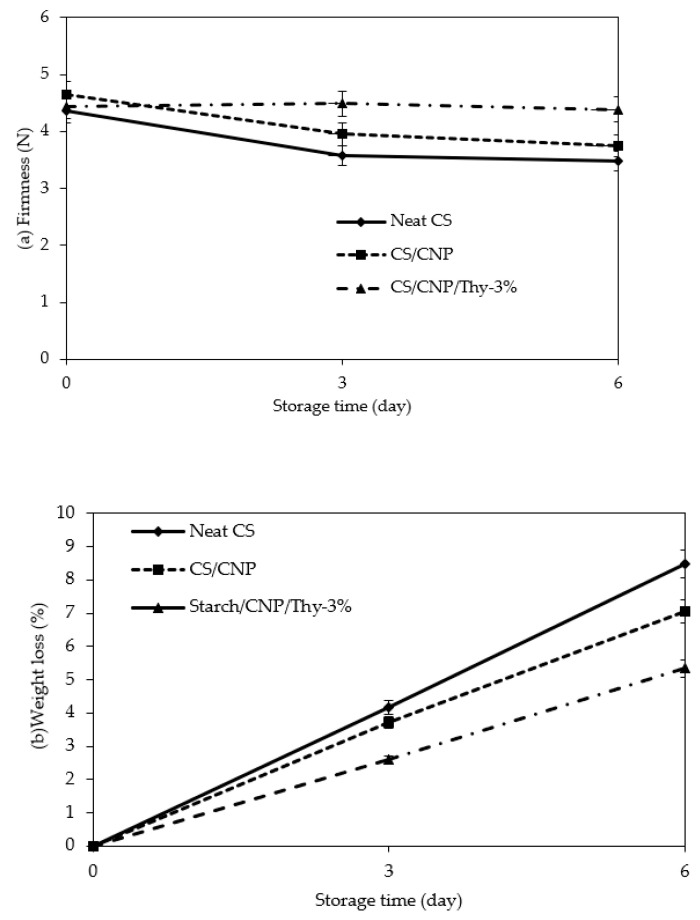
(**a**) Firmness and (**b**) percentage weight loss of cherry tomatoes packaged with neat CS films, CS/CNP bio-nanocomposite films, and CS/CNP/Thy 3% bio-nanocomposite films during 6 days’ storage at ambient condition.

To demonstrate the application of the developed films as food packaging, the films were used to packed cherry tomatoes and the shelf life of the cherry was determined in terms of firmness, weight loss, and observation of mold growth. Figure 6a shows the firmness of the cherry tomatoes packaged with different types of films over 6 days of storage at ambient temperature. In general, the firmness seems to decrease with the increase in the number of storage days for all the films because the cherry tomatoes underwent the ripening process during the storage. Abiso et al. [45] explained that fruit firmness decreased due to the ripening process that occurs and the softening of vegetative tissues that are usually caused by catabolism of cell wall polysaccharides (hemicellulose). The hemicelluloses and pectin became more soluble, which caused disruption and loosening of the cell walls resulting in the softness of food during ripening. In addition, change in the firmness of fresh products may occur due to loss of moisture through transpiration. The cherry tomatoes stored in the neat CS film, which acted as control, exhibited the maximum declination of firmness throughout the storage, which was 20% of the initial firmness, followed by cherry tomatoes stored in CS/CNP bio-nanocomposite films, which was 19% reduction in the initial firmness, and 1.4% reduction in the initial firmness for cherry tomatoes stored in CS/CNP/Thy 3% bio-nanocomposite films. 

The firmness of cherry tomatoes stored in CS/CNP/Thy-3% bio-nanocomposite films was maintained throughout the storage period compared to neat CS films and CS/CNP bio-nanocomposite films. This was due to the presence of thymol, a volatile antimicrobial agent that was released into the packaging, thus minimizing tissue softening and helping firmness retention during storage. Apart from that, the WVP of CS/CNP/Thy-3% bio-nanocomposite films was also low, thus reducing the loss of moisture through transpiration and maintaining the firmness of cherry tomatoes. Amal et al. [46], in their work, found that the highest values of firmness at the end of the storage period were achieved for strawberries coated with soy or gluten plus thymol. Correa-Pacheco et al. [47] also reported the same trend of results in which the avocado coated with the chitosan-thyme essential oil nanoparticle edible coating had higher fruit firmness compared to that of the untreated. Rahimi et al. [48] found that chitosan combined with thymol positively affected the retention of firmness in peach fruits by minimizing water loss and fruit senescence and reducing cell wall degradation by the action of inhibition of microbial propagation. In addition, Qin et al. [49] found that hot peppers stored in PLA/PLC/Thy films could maintain the ripening process at a slow rate due to the addition of thymol. The natural volatile compounds of thymol present in the headspace of food packaging can inhibit pathogen growth [50], thus helping to retain fruit firmness. Meanwhile, the firmness reduction in cherry tomatoes packed with CS/CNP bio-nanocomposite films was slightly lower (19%) than that of neat CS films (20%) due to the existence of CNP, which is also an antimicrobial agent. Nonetheless, CNP requires the food to be in direct contact with it before it can act as an antimicrobial agent, thus the reduction in firmness was much higher for CS/CNP compared to CS/CNP/Thy-3% bio-nanocomposite films.

The weight loss of fruits during storage is usually caused by water loss due to fruit transpiration [51]. Figure 6b shows that the percentage weight loss (%) of cherry tomatoes is directly proportional to the storage time (day) for all the samples. Cherry tomatoes packaged in CS/CNP bio-nanocomposite films exhibited a lower percentage of weight loss compared to that packaged in neat CS film due to the low WVP of CS/CNP bio-nanocomposite films as discussed in Section 3.3. The existence of CNP that occupied the empty spaces of the neat starch films matrix formed a tortuous path within the starch matrix. Thus, it was harder for water molecules to permeate due to the relatively compact structure of the films [52]. CNP was able to form a water barrier between the fruit and the external environment, thus minimizing the external transfer of water [51]. The same trend of the result was reported by Medina et al. [51], whereby cherry tomatoes and blueberries coated with chitosan thymol nanoparticles that were packed in clamshells exhibited a lower percentage of weight loss compared to the control film.

On the other hand, cherry tomatoes that were packed in CS/CNP/Thy-3% bio-nanocomposite films had the lowest weight loss, whereby the weight loss was approximately 3.1% and 1.8% lower than that were packed in neat CS films and CS/CNP bio-nanocomposite films on days 6, respectively. Despite the slightly higher WVP value compared to the CS/CNP bio-nanocomposite films, the weight loss of cherry tomatoes that were packed in CS/CNP/Thy-3% bio-nanocomposite films was still slightly lower than that of CS/CNP bio-nanocomposite films. A study carried out by Sun et al. [53] stated that the essential oil has hydrophobic traits which can reduce moisture loss of the food. A study carried out by Choi et al. [54] suggested that hydrophobic essential oils such as oregano and bergamot essential oils in hydroxypropyl methylcellulose were good water barriers to reduce moisture evaporation which directly causes the weight loss of fruit. Badawy et al. [55] reported the same trend of result whereby the incorporation of thymol into the film-forming solution resulted in a slightly positive effect on the weight loss reduction in strawberries packaged with the gelatin/chitosan films. A treatment of strawberries with thymol also showed a similar effect, whereby the strawberries exhibit the lowest weight loss after storage for 15 days at 0 °C [46]. Hence, thymol was proven to be an effective phenolic compound to minimize the dehydration of fruit and caused delay in the reduction in weight loss percentage. 

The effectiveness of the developed films as potential food packaging material was also evaluated by putting the films in direct contact with sliced cherry tomatoes and visually observed the occurrence of mold growth on the sliced cherry tomatoes with time. Table 3 shows the appearance of the sliced cherry tomato samples from day 0 until day 7. It was found that mold growth on sliced cherry tomato samples that were in direct contact with the CS/CNP bio-nanocomposite films (b) was slower than that of neat CS films (a). The same observation was reported by Lustriane et al. [56], whereby the incorporation of chitosan and CNP into the coating of the banana was found to retard the decay and maintain the quality of the banana. Xing et al. [57] stated that chitosan coating enriched with antimicrobials exhibited excellent inhibition on the growth of bacteria, yeast, and molds, whereas it can retard the growth of microbes on the food. A study conducted by Shapi’i et al. [58] found that starch/CNP films exhibit the potential as antimicrobial packaging. They found that cherry tomatoes packed with starch/CNP films exhibited the lowest mold growth due to the tiny size of CNP that can inhibit microbial growth.

Moreover, Table 3 shows that the CS/CNP/Thy-1.5% bio-nanocomposite films (c) exhibited satisfactory results in delaying the mold growth until day 6 of storage compared to CS/CNP bio-nanocomposite films (b). The incorporation of thymol as an antimicrobial agent helped to further slow down the growth of mold on sliced cherry tomatoes. A study by Medina et al. [51] reported that chitosan thymol nanoparticles had better efficiency in inhibiting mold growth compared to only CNP due to the addition of thymol, which contributed to more antimicrobial contents. Table 3 also presented that sliced cherry tomatoes in direct contact with CS/CNP/Thy-3% bio-nanocomposite films (d) have a consistent appearance with no microbial/mold growth until the end of the storage period. This result demonstrated that a 3*w/w*% concentration of thymol incorporated in the films was the optimum concentration to inhibit microbial growth and prolong the shelf life of the cherry tomatoes. The release of thymol from CS/CNP/Thy-3% bio-nanocomposite films attenuated the reactive oxygen species and produced a suitable atmosphere that could reduce the respiration rate and fungal growth with minimal alteration of organoleptic properties and increase the shelf life of cherry tomatoes. The microbial mode of action of thymol has been postulated as a disruption of cellular membrane functions and interference of active sites of enzymes and cellular metabolism [59]. This causes the change in the permeability of membranes of the microbes for cations and alters the ion gradients that lead the microbe cell to destruct and, consequently, to cell death. 

Nonetheless, it was found in this study that sliced cherry tomatoes in direct contact with the CS/CNP/Thy-4.5% bio-nanocomposite films started to exhibit mold growth on the surface from day 3. The 4.5*w/w*% of thymol concentration might be too high, which interfered with the gas diffusion through the films, and the lack of gas diffusion might generate heat and anaerobic conditions, which will lead to mold growth [56]. Additionally, the problem may be due to the difficulty of uniform dispersion of the hydrophobic thymol that caused non-homogenized film-forming solutions. Moreover, thymol had limited water solubility, thus the undissolved thymol due to the concentration above the solubility limit might impact the quality and antimicrobial availability [60]. Thus, the incorporation of the optimum concentration of thymol (3*w/w*%) in the films exhibits the potential to increase the shelf life of cherry tomatoes during storage as presented from the satisfactory results in the inhibition of the mold growth of cherry tomatoes.

## 4. Conclusions

CS/CNP/Thy bio-nanocomposite films were successfully developed, characterized, and demonstrated as potential active food packaging material. It was found that the changes in opacity and color properties of the films with the addition of thymol were only minimal and almost non-noticeable. The addition of thymol to the films reduced the mechanical properties (TS, EAB, and YM) of the films due to the plasticizing effect of thymol, but the increment of concentrations of thymol did not significantly affect the mechanical properties due to the low concentrations of thymol (≤4.5*w/w*%). It was also found that the addition of 1.5*w/w*% thymol to CS/CNP bio-nanocomposite films increased the WVP but the WVP decreased with the increment of thymol concentrations due to the hydrophobic nature of thymol which disrupted the hydrophilic/hydrophobic balance of the film and its ability to limit the water vapor penetration through the film. In addition, the CS/CNP/Thy 3% bio-nanocomposite films were efficient in maintaining the firmness and reducing the weight loss of cherry tomatoes compared to neat CS films and CS/CNP bio-nanocomposite films during 6 days of storage. The CS/CNP/Thy 3% bio-nanocomposite films also demonstrated the potential to lengthen the shelf life of cherry tomatoes by inhibiting the mold growth on cherry tomatoes during 7 days of storage. The potential of the CS/CNP/Thy bio-nanocomposite films as active food packaging material shall be further explored and further work shall be conducted on the structure and morphology; thermal; and antimicrobial properties of the films.

## Figures and Tables

**Table 1:** Formulation of CS/CNP/Thy bio-nanocomposite film forming solutions.

Percentage Weight of Thymol/Weight of Starch (*w/w*%)	Weight of Chitosan (g)	Weight of Thymol (g)	Weight of Tween 80 (µL)	Weight of TPP (g)
0	0.45	0	0	0.09
1.5	0.45	0.045	275	0.09
3.0	0.45	0.09	275	0.09
4.5	0.45	0.135	275	0.09

**Table 2:** Optical properties of starch/CNP/Thy bio-nanocomposite films incorporated with different concentrations of thymol. The data are reported a mean ± SD, n = 3 and *p* < 0.05.

Concentration of Thymol (% *w/w*)	L*	a*	b*	Total Color Difference (ΔE)	Opacity (A_600_/mm)
0%	93.77 ± 0.14 ^a^	−1.10 ± 0.02 ^ab^	3.74 ± 0.19 ^b^	0	0.80 ± 0.04 ^b^
1.5%	92.99 ± 0.03 ^b^	−1.04 ± 0.05 ^a^	3.60 ± 0.21 ^b^	0.81 ± 0.03 ^b^	0.81 ± 0.02 ^b^
3.0%	92.95 ± 0.10 ^b^	−1.12 ± 0.07 ^ab^	3.62 ± 0.13 ^b^	0.84 ± 0.10 ^b^	0.87 ± 0.01 ^b^
4.5%	92.54 ± 0.05 ^c^	−1.19 ± 0.03 ^b^	4.24 ± 0.06 ^a^	1.33 ± 0.03 ^a^	1.06 ± 0.07 ^a^

Values in the same column showing the same superscripts are not significantly different (*p* > 0.05).

**Table 3:** Appearance of sliced cherry tomatoes in direct contact with (a) neat CS film; (b) CS/CNP bio-nanocomposite film; (c) CS/CNP/Thy 1.5% bio-nanocomposite film; (d) CS/CNP/Thy 3% bio-nanocomposite film; (e) CS/CNP/Thy 4.5% bio-nanocomposite film.

	Day 0	Day 1	Day 2	Day 3	Day 4	Day 5	Day 6	Day 7
(**a**)	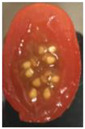	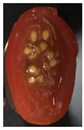	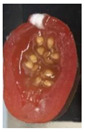	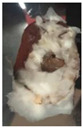	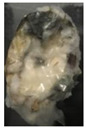	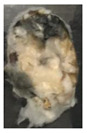	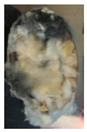	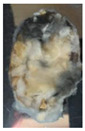
(**b**)	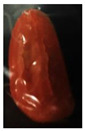	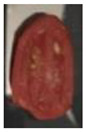	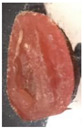	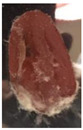	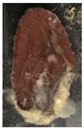	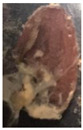	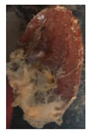	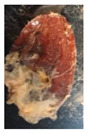
(**c**)	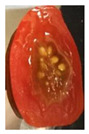	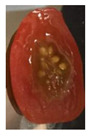	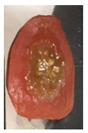	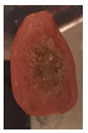	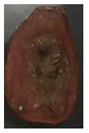	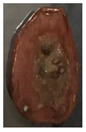	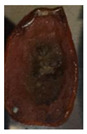	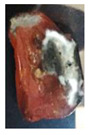
(**d**)	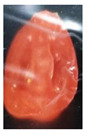	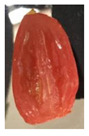	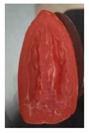	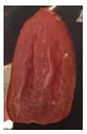	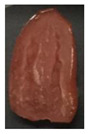	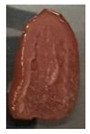	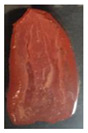	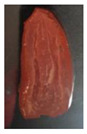
(**e**)